# Supplementary material for: Differences in metalloproteinases and their tissue inhibitors in the cerebrospinal fluid are associated with delirium
Source: Commun Med (Lond). 2024 Jun 27;4:124. doi: 10.1038/s43856-024-00558-z (PMC11211460; doi:10.1038/s43856-024-00558-z)
Supplement: Supplementary file 2 — Supplementary Information [file 43856_2024_558_MOESM2_ESM.docx]

Differences in metalloproteinases and their tissue inhibitors in the cerebrospinal fluid are associated with delirium

# Supplementary Information

Supplementary Table 1. Characteristics of the hip fracture patients stratified by dementia-status.

|  | **All** | **No dementia** | **Dementia** | ***P*** |
| --- | --- | --- | --- | --- |
| **N** | 279 | 161 | 118 |  |
| **Age** | 84 (74; 89) | 80 (71; 87) | 86 (81; 90) | 9.4*10^-6^ |
| **Female sex, n(%)** | 192 (68.8) | 113 (70.2) | 79 (67.0) | 0.56 |
| **Delirium** | 140 (50.0) | 41 (29.3) | 99 (83.9) | 5.2*10^-22^ |
| **Dementia** | 118 (42.1) | 0 (0) | 118 (100) |  |
| **IQCODE** | 3.3 (3; 4.2) | 3.1 (3; 3.2) | 4.4 (3.9; 4.9) | 2.7*10^-14^ |
| **ASA III-IV, n(%)** | 145 (52.0) | 66 (41.0) | 80 (67.8) | 3.6*10^-7^ |
| **MMP-2 (ng/mL)** | 59.2 (49.3; 69.4) | 58.0 (47.9; 65.2) | 62.3 (52.3; 73.1) | 0.01 |
| **MMP-3 (pg/mL)** | 227 (154; 335) | 223 (152; 316) | 238 (164; 360) | 0.15 |
| **MMP-10 (pg/mL)** | 24.2 (13.5; 41.3) | 20.4 (10.3; 34.3) | 33.7 (19.2; 45.7) | 9.4*10^-6^ |
| **MMP-12 (pg/mL)** | 3.6 (0.4; 6.7) | 3.6 (0.4; 6.7) | 3.6 (0.4; 7.8) | 0.58 |
| **TIMP-1 (ng/mL)** | 87.3 (66.3; 110.0) | 85.3 (61.7; 104.5) | 91.3 (71.9; 116.6) | 0.03 |
| **TIMP-2 (ng/mL)** | 80.5 (69.0; 93.7) | 78.8 (67.9; 89.8) | 83.7 (70.8; 99.0) | 0.05 |
| **TIMP-3 (ng/mL)** | 15.7 (14.4; 17.0) | 15.7 (14.1; 16.7) | 15.7 (14.5; 17.4) | 0.15 |
| **TIMP-4 (ng/mL)** | 1.6 (1.3; 1.9) | 1.6 (1.3; 2.0) | 1.5 (1.2; 1.8) | 0.07 |

Data is presented as median (quartile 1; quartile 3) unless otherwise indicated. *P* value is for comparison between patients with and without delirium using Mann Whitney U test (continuous variables) or χ^2^-test (categorical variables). **Abbreviations:** ASA; American Society of Anesthesiologists physical status classification; IQCODE: Informant Questionnaire on Cognitive Decline in the Elderly; MMP: matrix metalloproteinase; TIMP: tissue inhibitor of matrix metalloproteinase

Supplementary Table 2. Characteristics of the hip fracture patients stratified by dementia and delirium status.

| **Without dementia** | | | | | |
| --- | --- | --- | --- | --- | --- |
|  | **All** | **No delirium** | **Delirium** |  |  |
|  |  |  | **All** | **Incident** | **Prevalent^b^** |
| **N** | 161 | 120 | 41 | 27 | 14 |
| **Age** | 80 (71; 87) | 76 (69; 85.5) | 87 (80; 91)^a^ | 87 (77; 91)^a^ | 87.5 (80; 94)^a^ |
| **Female sex, n (%)** | 113 (70.2) | 86 (71.7) | 27 (65.9) | 18 (66.7) | 9 (64.3) |
| **Delirium, n (%)** | 41 (25.5) | 0 (0) | 100 (100) | 100 (100) | 100 (100) |
| **IQCODE** | 3.1 (3; 3.2) | 3 (3; 3.2) | 3.2 (3.1; 3.3)^a^ | 3.2 (3.1; 3.3)^a^ | 3.2 (3.1; 3.3)^a^ |
| **ASA III-IV, n(%)** | 66 (41.0) | 40 (33.3) | 26 (63.4)^a^ | 17 (63.0)^a^ | 9 (64.3) |
| **Pre-fracture dementia** | | | | | |
|  | **All** | **No delirium** | **Delirium** |  |  |
|  |  |  | **All** | **Incident** | **Prevalent^b^** |
| **N** | 118 | 19 | 99 | 46 | 53 |
| **Age** | 86 (81; 90) | 82 (72; 87) | 86 (82; 91)^a^ | 86 (81; 91)^a^ | 87 (82; 91)^a^ |
| **Female sex, n (%)** | 79 (67.0) | 13 (68.4) | 66 (66.7) | 29 (63.0) | 37 (69.8) |
| **Delirium, n (%)** | 99 (83.9) | 0 (0) | 100 (100) | 100 (100) | 100 (100) |
| **IQCODE** | 4.4 (3.9; 4.9) | 4.1 (3.8; 4.5) | 4.5 (3.9; 4.9) | 4.4 (3.9; 4.8) | 4.6 (3.9; 5)^a^ |
| **ASA III-IV, n(%)** | 80 (67.8) | 14 (73.7) | 66 (66.7) | 27 (58.7) | 39 (73.6) |

Data is presented as median (quartile 1; quartile 3) unless otherwise indicated. *P* value is for comparison between patients with and without delirium using Mann Whitney U test (continuous variables) or χ^2^-test (categorical variables). ^a,^ significantly different vs. no delirium; ^b^ no significant differences vs. incident delirium. **Abbreviations:** ASA; American Society of Anesthesiologists physical status classification; IQCODE: Informant Questionnaire on Cognitive Decline in the Elderly.

Supplementary Table 3. Matrix metalloproteinase and tissue inhibitor of matrix metalloproteinase levels in hip fracture patients by dementia and delirium status.

| **Without dementia** | | | | | |
| --- | --- | --- | --- | --- | --- |
|  | **All** | **No delirium** | **Delirium** |  |  |
|  |  |  | **All** | **Incident** | **Prevalent** |
| **N** | 161 | 120 | 41 | 27 | 14 |
| **MMP-2 (ng/mL)** | 58.0 (47.9; 65.2) | 57.2 (46.5; 63.9) | 60.4 (53.7; 73.8) | 60.3 (53.7; 69.7) | 63.9 (51.4; 86.2) |
| **MMP-3 (pg/mL)** | 223 (152; 316) | 201 (148; 284) | 301 (202; 364)* | 279 (149; 367) | 317 (247; 360)* |
| **MMP-10 (pg/mL)** | 20.4 (10.3; 34.3) | 17.3 (9.7; 28.6) | 33.1 (14.7; 49.5)* | 31.8 (12.8; 53.3) | 38.1 (19.2; 48.2)* |
| **MMP-12 (pg/mL)** | 3.6 (0.4; 6.7) | 3.6 (0.4; 7.2) | 3.6 (0.4; 6.7) | 3.6 (0.4; 6.7) | 3.6 (0.4; 4.6) |
| **TIMP-1 (ng/mL)** | 85.3 (61.7; 105) | 83.1 (60.5; 101) | 91.9 (69.5; 121) | 91.9 (70.1; 128) | 87.1 (60.8; 117) |
| **TIMP-2 (ng/mL)** | 78.8 (67.9; 89.8) | 77.1 (66.4; 87.3) | 87.7 (69.8; 99.5)* | 87.9 (69.8; 105) | 83.7 (69.0; 96.1) |
| **TIMP-3 (ng/mL)** | 15.7 (14.1; 16.7) | 15.6 (14.1; 16.7) | 15.9 (14.4; 17.5) | 16.1 (13.1; 18.1) | 15.8 (13.3; 16.8) |
| **TIMP-4 (ng/mL)** | 1.6 (1.3; 2.0) | 1.6 (1.4; 2.0) | 1.4 (1.2; 1.9) | 1.5 (1.2; 1.9) | 1.4 (1.2; 1.9) |
| **Pre-fracture dementia** | | | | | |
|  | **All** | **No delirium** | **Delirium** |  |  |
|  |  |  | **All** | **Incident** | **Prevalent** |
| **N** | 118 | 19 | 99 | 46 | 53 |
| **MMP-2 (ng/mL)** | 62.3 (52.3; 73.1) | 56.6 (51.0; 71.4) | 62.4 (52.4; 73.2) | 62.3 (49.3; 68.4) | 63.6 (54.5; 78.5) |
| **MMP-3 (pg/mL)** | 238 (164; 360) | 235 (146; 400) | 238 (168; 356) | 220 (139; 371) | 248 (204; 333) |
| **MMP-10(pg/mL)** | 33.7 (19.2; 45.7) | 35.6 (17.9; 57.7) | 31.2 (19.2; 45.7) | 25.8 (17.9; 44.4) | 33.7 (21.7; 45.7) |
| **MMP-12 (pg/mL)** | 3.6 (0.4; 7.8) | 5.7 (1.5; 8.8) | 3.6 (0.4; 7.8) | 3.6 (1.5; 8.8) | 2.5 (0.4; 5.7) |
| **TIMP-1 (ng/mL)** | 91.3 (71.9; 117) | 77.6 (54.7; 109) | 93.2 (74.4; 121) | 91.2 (75.6; 112) | 97.5 (70.3; 123) |
| **TIMP-2 (ng/mL)** | 83.7 (70.8; 99.0) | 74.6 (60.6; 95.9) | 85.4 (71.1; 99.1) | 84.5 (72.3; 94.7) | 87.3 (70.9; 101) |
| **TIMP-3 (ng/mL)** | 15.7 (14.4; 17.4) | 15.2 (14.2; 17.6) | 15.9 (14.5; 17.4) | 15.8 (14.8; 17.3) | 16.1 (14.0; 17.4) |
| **TIMP-4 (ng/mL)** | 1.5 (1.2; 1.8) | 1.6 (1.2; 2.0) | 1.5 (1.2; 1.8) | 1.4 (1.2; 1.8) | 1.5 (1.3; 1.8) |

Data is presented as median (quartile 1; quartile 3) unless otherwise indicated. *significantly different vs. no delirium, Mann-Whitney U test, *P* < 0.01; there were no significant differences for prevalent vs. incident delirium. **Abbreviations:** MMP: matrix metalloproteinase; TIMP: tissue inhibitor of matrix metalloproteinase

Supplementary Table 4: Associations of CSF MMP/TIMPs with restlessness, hallucinations and illusions in the delirium cohort.

|  | **Restlessness (n = 128)** | | **Hallucinations (n = 119)** | | **Illusions (n = 121)** |  |
| --- | --- | --- | --- | --- | --- | --- |
|  | **Odds ratio (95% CI)** | ***P*** | **Odds ratio (95% CI)** | ***P*** | **Odds ratio (95% CI)** | ***P*** |
| **MMP-2** | 0.98 (0.96; 1.01) | 0.14 | 1.00 (0.98; 1.03) | 0.55 | 1.02 (1.00; 1.05) | 0.08 |
| **MMP-3** | 0.99 (1.00; 1.00) | 0.39 | 1.00 (1.00; 1.00) | 0.25 | 1.00 (1.00; 1.00) | 0.75 |
| **MMP-10** | 0.99 (0.97; 1.00) | 0.10 | 0.99 (0.97; 1.01) | 0.20 | 1.00 (0.99; 1.02) | 0.60 |
| **MMP-12** | 0.96 (0.88; 1.06) | 0.43 | 0.94 (0.83; 1.05) | 0.28 | 1.06 (0.94; 1.16) | 0.37 |
| **TIMP-1** | 0.99 (0.99; 1.01) | 0.63 | 1.00 (0.99; 1.02) | 0.49 | 1.00 (0.99; 1.02) | 0.63 |
| **TIMP-2** | 1.00 (0.98; 1.01) | 0.65 | 1.00 (0.98; 1.03) | 0.57 | 1.00 (0.99; 1.03) | 0.38 |
| **TIMP-3** | 0.92 (0.76; 1.11) | 0.37 | 1.00 (0.80; 1.25) | 0.99 | 1.04 (0.83; 1.30) | 0.73 |
| **TIMP-4** | 1.22 (0.56; 2.66) | 0.62 | 0.66 (0.25; 1.78) | 0.41 | 0.89 (0.33; 2.40) | 0.82 |

Adjusted logistic regression models predicting the odds of restlessness, hallucinations and illusions in the delirium cohort. Models are adjusted for sex, age, and presence/absence of dementia (IQCODE ≥ 3.44 = dementia). **Abbreviations:** CI: confidence interval; IQCODE: Informant Questionnaire on Cognitive Decline in the Elderly; MMP: matrix metalloproteinase; TIMP: tissue inhibitor of matrix metalloproteinase

Supplementary Table 5: Associations between CSF MMP/TIMP levels and 1-year survival after hip fracture.

|  | **Univariate analyses** | | **Multivariate analyses** | |
| --- | --- | --- | --- | --- |
|  | **Hazard ratio** | ***P*** | **Hazard ratio** | ***P*** |
| **MMP-2** | 1.02 (1.01; 1.03) | 0.001 | 1.00 (0.99; 1.02) | 0.75 |
| **MMP-3** | 1.001 (1.00; 1.002) | 0.03 | 1.00 (1.00; 1.00) | 0.83 |
| **MMP-10** | 1.01 (1.00; 1.01) | 0.005 | 1.00 (0.99; 1.01) | 0.48 |
| **MMP-12** | 0.96 (0.90; 1.02) | 0.20 | 0.97 (0.90; 1.04) | 0.34 |
| **TIMP-1** | 1.01 (1.01; 1.02) | 0.001 | 1.00 (1.00; 1.01) | 0.31 |
| **TIMP-2** | 1.03 (1.02; 1.04) | < 0.001 | 1.01 (1.00; 1.02) | 0.05 |
| **TIMP-3** | 1.20 (1.05; 1.38) | 0.007 | 1.05 (0.92; 1.21) | 0.47 |
| **TIMP-4** | 0.70 (0.41; 1.20) | 0.20 | 0.79 (0.45; 1.38) | 0.41 |

Cox regression conducted on 278 hip fracture patients with 59 failures (deaths) and censoring after 365 days following admission. Multivariate analyses are adjusted for delirium, age, sex and dementia. **Abbreviations:** MMP: matrix metalloproteinase; TIMP: tissue inhibitor of matrix metalloproteinase

Supplementary Table 6. Characteristics of the hip fracture patients stratified by sex.

|  | **All** | **Women** | **Men** | ***P*** |
| --- | --- | --- | --- | --- |
| **N** | 279 | 192 | 87 |  |
| **Age** | 84.0 (74.0; 89.0) | 82.0 (74.0; 89.0) | 85.0 (74.0; 89.0) | 0.56 |
| **Delirium** | 139 (49.8) | 93 (48.8) | 47 (54.0) | 0.39 |
| **Dementia** | 118 (42.3) | 79 (41.1) | 39 (44.8) | 0.56 |
| **IQCODE** | 3.3 (3.0; 4.2) | 3.3 (3.0; 4.0) | 3.3 (3.0; 4.4) | 0.66 |
| **ASA** **III-IV, n(%)** | 146 (52.3) | 92 (47.9) | 54 (62.1) | 0.04 |
| **MMP-2 (ng/mL)** | 59.2 (49.3; 69.4) | 58.6 (49.2; 65.7) | 63.0 (49.3; 79.3) | 0.02 |
| **MMP-3 (pg/mL)** | 227 (154; 335) | 206 (145; 300) | 275 (205; 384) | 5.6*10^-5^ |
| **MMP-10 (pg/mL)** | 24.2 (13.5; 41.3) | 22.3 (12.8; 38.8) | 29.9 (15.4; 59.6) | 0.006 |
| **MMP-12 (pg/mL)** | 3.6 (0.4; 6.7) | 4.1 (0.4; 7.8) | 2.5 (0.4; 6.7) | 0.31 |
| **TIMP-1 (ng/mL)** | 87.3 (66.3; 110) | 85.2 (63.9; 105) | 96.3 (68.0; 122) | 0.05 |
| **TIMP-2 (ng/mL)** | 80.5 (69.0; 93.7) | 77.1 (67.9; 89.3) | 87.3 (71.1; 105) | 0.001 |
| **TIMP-3 (ng/mL)** | 15.7 (14.4; 17.0) | 15.6 (14.3; 16.8) | 16.1 (14.4; 17.5) | 0.08 |
| **TIMP-4 (ng/mL)** | 1.6 (1.3; 1.9) | 1.6 (1.3; 2.0) | 1.5 (1.2; 1.8) | 0.35 |

Data is presented as median (quartile 1; quartile 3) unless otherwise indicated. *P* value is for comparison between patients with and without delirium using Mann Whitney U test (continuous variables) or χ^2^-test (categorical variables). **Abbreviations:** ASA; American Society of Anesthesiologists physical status classification; IQCODE: Informant Questionnaire on Cognitive Decline in the Elderly; MMP: matrix metalloproteinase; TIMP: tissue inhibitor of matrix metalloproteinase

Supplementary Table 7. Correlations with age across the CU group, the AD group and the hip fracture patients.

|  | **All** | **CU group** | **AD group** | **Hip fracture patients** |
| --- | --- | --- | --- | --- |
| **N** | 497 | 107 | 111 | 279 |
| **MMP-2** | 0.58 | 0.34 | 0.32 | 0.44 |
| **MMP-3** | 0.22 | -0.001 | 0.16 | 0.34 |
| **MMP-10** | 0.28 | 0.05 | 0.15 | 0.39 |
| **MMP-12** | 0.11 | 0.09 | -0.06 | 0.004 |
| **TIMP-1** | 0.54 | 0.39 | 0.33 | 0.42 |
| **TIMP-2** | 0.45 | 0.29 | 0.25 | 0.41 |
| **TIMP-3** | 0.12 | 0.24 | 0.10 | 0.31 |
| **TIMP-4** | 0.04 | -0.02 | 0.09 | 0.10 |

Spearman’s rho for each MMP and TIMP with age across the three cohorts.
**Abbreviations:** AD: Alzheimer’s disease; CU: cognitively unimpaired; MMP: matrix metalloproteinase; TIMP: tissue inhibitor of matrix metalloproteinase

Supplementary Table 8. Correlations with CSF Aβ_42_, p-tau_181_ and NFL in the hip fracture patients.

|  | **Aβ_42_** | **P-tau_181_** | **NFL** |
| --- | --- | --- | --- |
| **MMP-2** | 0.08 | 0.39 | 0.64 |
| **MMP-3** | 0.12 | 0.36 | 0.32 |
| **MMP-10** | 0.00 | 0.46 | 0.52 |
| **MMP-12** | -0.06 | -0.03 | -0.06 |
| **TIMP-1** | -0.02 | 0.33 | 0.46 |
| **TIMP-2** | -0.04 | 0.28 | 0.50 |
| **TIMP-3** | 0.00 | 0.26 | 0.42 |
| **TIMP-4** | 0.41 | 0.35 | -0.07 |

Data presented is Spearman’s rho. **Abbreviations:** Aβ: amyloid-β; MMP: matrix metalloproteinase; NFL: neurofilament light; p-tau: phosphorylated tau; TIMP: tissue inhibitor of matrix metalloproteinase
