# Supplementary material for: Differences in metalloproteinases and their tissue inhibitors in the cerebrospinal fluid are associated with delirium
Source: Commun Med (Lond). 2024 Jun 27;4:124. doi: 10.1038/s43856-024-00558-z (PMC11211460; doi:10.1038/s43856-024-00558-z)
Supplement: Supplementary file 3 — Description of Additional Supplementary Files [file 43856_2024_558_MOESM3_ESM.pdf]

Description of additional supplementary files

File name: Supplementary Data 1

File description: Numerical data underlying Figure 1 (Sheet 1) and Figure 2 (Sheet 2) are provided as supplementary data.
