# Supplementary material for: Differences in metalloproteinases and their tissue inhibitors in the cerebrospinal fluid are associated with delirium
Source: Commun Med (Lond). 2024 Jun 27;4:124. doi: 10.1038/s43856-024-00558-z (PMC11211460; doi:10.1038/s43856-024-00558-z)
Supplement: Supplementary file 5 — Reporting Summary [file 43856_2024_558_MOESM5_ESM.pdf]

Corresponding author(s): Mari Aksnes, PhD

Last updated by author(s): Apr 22, 2024

Reporting Summary

Nature Portfolio wishes to improve the reproducibility of the work that we publish. This form provides structure for consistency and transparency in reporting. For further information on Nature Portfolio policies, see our Editorial Policies and the Editorial Policy Checklist.

Statistics

For all statistical analyses, confirm that the following items are present in the figure legend, table legend, main text, or Methods section.

n/a	Confirmed	
☐	☒	The exact sample size (*n*) for each experimental group/condition, given as a discrete number and unit of measurement
☐	☒	A statement on whether measurements were taken from distinct samples or whether the same sample was measured repeatedly
☐	☒	The statistical test(s) used AND whether they are one- or two-sided<br>*Only common tests should be described solely by name; describe more complex techniques in the Methods section.*
☐	☒	A description of all covariates tested
☐	☒	A description of any assumptions or corrections, such as tests of normality and adjustment for multiple comparisons
☐	☒	A full description of the statistical parameters including central tendency (e.g. means) or other basic estimates (e.g. regression coefficient) AND variation (e.g. standard deviation) or associated estimates of uncertainty (e.g. confidence intervals)
☐	☒	For null hypothesis testing, the test statistic (e.g. *F*, *t*, *r*) with confidence intervals, effect sizes, degrees of freedom and *P* value noted<br>*Give P values as exact values whenever suitable.*
☒	☐	For Bayesian analysis, information on the choice of priors and Markov chain Monte Carlo settings
☒	☐	For hierarchical and complex designs, identification of the appropriate level for tests and full reporting of outcomes
☐	☒	Estimates of effect sizes (e.g. Cohen's *d*, Pearson's *r*), indicating how they were calculated

Our web collection on statistics for biologists contains articles on many of the points above.

Software and code

Policy information about availability of computer code

Data collection	No software was used.
Data analysis	Statistical analyses were performed using STATA version 16.1. Data visualisations were created in R4.1.1. using RStudio version 2023.06.0 +421.

For manuscripts utilizing custom algorithms or software that are central to the research but not yet described in published literature, software must be made available to editors and reviewers. We strongly encourage code deposition in a community repository (e.g. GitHub). See the Nature Portfolio guidelines for submitting code & software for further information.

Data

Policy information about availability of data

All manuscripts must include a data availability statement. This statement should provide the following information, where applicable:

- Accession codes, unique identifiers, or web links for publicly available datasets
- A description of any restrictions on data availability
- For clinical datasets or third party data, please ensure that the statement adheres to our policy

The datasets analysed in the current study are not publicly available due to legal restrictions concerning the disclosure of sensitive personal data. The clinical data for the memory clinic patients may be requested from the Norwegian Registry of Persons Assessed for Cognitive Symptoms (NorCog) at e-mail: post@aldringoghelse.no. The clinical data for the cognitively unimpiared controls and hip fracture patients, as well as results of the MMP/TIMP analysis for all

cohorts, are available upon reasonable request to the corresponding author. All data availability is dependent on the approval from the Regional Committee for Ethics in Medical Research in Norway, contact at e-mail: post@helseforskning.etikkom.no

Human research participants

Policy information about studies involving human research participants and Sex and Gender in Research.

Reporting on sex and gender	In the current study, sex was determined based on medical journals. We had no a priori hypotheses connected to sex differences. In the hip fracture cohort, there was no statistically significant difference in the proportion of men and women in the no delirium versus delirium group. Sex was not a significant predictor of odds of delirium in adjusted analysis. We have reported results stratified by sex for the hip fracture cohort in the Results section and in detail in the Supplementary Information (Supplementary Table 5).
Population characteristics	We included three groups of human research participants: 280 hip fracture patients, 107 cognitively unimpaired persons aged 65 years or older and 111 patients with Alzheimer's disease dementia.<br>The hip fracture patients had a median age of 84 years (25th percentile: 74 years; 75th percentile: 89 years). The group consisted of 192 women and 87 men. 50 % of the hip fracture patients experienced delirium and 42.1 % had pre-delirium dementia.<br>The cognitively unimpaired individuals had a median age of 71 years (25th percentile: 67 years; 75th percentile 76 years). The group consisted of 48 women and 59 men. There was no presence of delirium or dementia in this group.<br>The Alzheimer's disease dementia patients had a median age of 71 years(25th percentile: 66 years; 75th percentile 75 years). The group consisted of 68 women and 43 men. All of the patients in this group had dementia and none of the patients had delirium.
Recruitment	Hip fracture patients were included from a multi-centre study at the Oslo University Hospital, Diakonhjemmet Hospital, Akershus University Hospital and Bærum Hospital in the Oslo region of Norway between 2016 and 2019. Cognitively unimpaired individuals were recruited from patients undergoing elective gynaecological, orthopaedic or urological surgery in spinal anaesthesia at Oslo University Hospital or Diakonhjemmet Hospital between 2012 and 2013. For this group, only individuals considered cognitively unimpaired at baseline were included.<br>Alzheimer's disease dementia patients were recruited from the Norwegian Registry of Persons Assessed for Cognitive Symptoms (NorCog). We included patients assessed at two Norwegian memory clinics, Oslo University Hospital and St. Olav University Hospital, between 2009 and 2018. All patients assessed at these memory clinics are asked to participate in NorCog.
Ethics oversight	The study was approved by the Regional Committee for Ethics in Medical Research in Norway (REC Central, #19337; REC South East, #12064, #6897).

Note that full information on the approval of the study protocol must also be provided in the manuscript.

Field-specific reporting

Please select the one below that is the best fit for your research. If you are not sure, read the appropriate sections before making your selection.

☒ Life sciences ☐ Behavioural & social sciences ☐ Ecological, evolutionary & environmental sciences

For a reference copy of the document with all sections, see nature.com/documents/nr-reporting-summary-flat.pdf

Life sciences study design

All studies must disclose on these points even when the disclosure is negative.

Sample size	From the hip fracture cohort, we included all patients that had CSF available in the biobank for analysis of the MMP and TIMP proteins. Cognitively unimpaired individuals were included from the Cognorm study, and we included all individuals that had CSF available in the biobank and were considered cognitively unimpaired at baseline in line with pre-set criteria. Alzheimer's disease patients were included from the NorCog register and we included all patients with CSF available in the biobank for MMP analysis that met the criteria for probable and possible dementia due to Alzheimer's disease and had pathological levels of the established CSF biomarkers for Alzheimer's disease (low CSF amyloid-beta42 and high CSF phosphorylated tau181).
Data exclusions	The proteins MMP-1, MMP-7, MMP-8, MMP-9 and MMP-13 were analysed but detectable in less than 50 % of samples and therefore excluded from further analysis.<br>One hip fracture patient had extreme values (> 15 standard deviations above the mean) of several MMPs and was excluded from further analysis. The rationale behind the exclusion is that this extreme value likely is due to measurement error or some other underlying disease process unrelated to dementia and delirium.
Replication	For the biochemical analysis, all samples were run in duplicate to control for consistency of the analysis and increase reproducibility.
Randomization	Patients were group according to their cognitive status and/or whether they experienced delirium. This allocation was not random as these are medical conditions that cannot be experimentally assigned. Relecant covariates were controlled for in analyses adjusted for sex, age and cognitive impairment.

Blinding	The investigators were not blinded to group allocation during data collection: data collection was performed by patient's physicians as part of a clinical evaluation and blinding was not possible. The personell performing the biochemical analysis were blinded to group allocation and patient information. The person performing statistical analysis was not blinded to group allocation; this was not possible due to distinct and recognisable differences in the population characteristics of the included groups (i.e. presence or abscence of dementia and delirium).

Reporting for specific materials, systems and methods

We require information from authors about some types of materials, experimental systems and methods used in many studies. Here, indicate whether each material, system or method listed is relevant to your study. If you are not sure if a list item applies to your research, read the appropriate section before selecting a response.

Materials & experimental systems

n/a	Involved in the study
☐	☒ Antibodies
☒	☐ Eukaryotic cell lines
☒	☐ Palaeontology and archaeology
☒	☐ Animals and other organisms
☒	☐ Clinical data
☒	☐ Dual use research of concern

Methods

n/a	Involved in the study
☒	☐ ChIP-seq
☒	☐ Flow cytometry
☒	☐ MRI-based neuroimaging

Antibodies

Antibodies used	MMPs and TIMPs were quantified by Eve Technologies using a 13-plex Discovery Assay(R): "Human MMP and TIMP Discovery Assay® Array for Cell Culture and non-blood samples (HMMP/TIMP-C,O)". This assay includes antibodies for the following biomarkers: MMP-1 (Collagenase 1), MMP-2 (Gelatinase A), MMP-3 (Stromelysin 1), MMP-7 (Matrilysin), MMP-8 (Collagenase 2), MMP-9 (Gelatinase B)*, MMP-10 (Matrix Metalloproteinase 10), MMP-12 (Macrophage Metalloelastase), MMP-13 (Collagenase 3)**, TIMP-1, TIMP-2, TIMP-3, TIMP-4. Supplier name, catalog number, clone name, and lot number are not provided by Eve Technologies. CSF Aβ42 and p-tau181 concentrations for the hip fracture cohort were measured using INNOTEST enzyme-linked immunosorbent assays (Fujirebio) at Sahlgrenska University Hospital (Mölndal, Sweden).
Validation	Details on validation for each primary antibody used in the Discovery Assay are not provided by Eve Technologies, however they are a widely used multiplex assaying service and the results of their assays are widely published. The INNOTEST enzyme-linked immunosorbent assays for CSF Aβ42 and p-tau181 are validated and widely used for Alzheimer's disease biomarker measurement.
